# Epidemic cycles driven by host behaviour

**DOI:** 10.1098/rsif.2014.0575

**Published:** 2014-10-06

**Authors:** Benjamin M. Althouse, Laurent Hébert-Dufresne

**Affiliations:** 1 Santa Fe Institute, Santa Fe, NM 87501, USA; 2 Département de Physique, de Génie Physique, et d'Optique, Université Laval, Québec (Québec), Canada G1V 0A6

**Keywords:** syphilis, network model, epidemic cycles, human behaviour

## Abstract

Host immunity and demographics (the recruitment of susceptibles via birthrate) have been demonstrated to be a key determinant of the periodicity of measles, pertussis and dengue epidemics. However, not all epidemic cycles are from pathogens inducing sterilizing immunity or are driven by demographics. Many sexually transmitted infections are driven by sexual behaviour. We present a mathematical model of disease transmission where individuals can disconnect and reconnect depending on the infectious status of their contacts. We fit the model to historic syphilis (*Treponema pallidum*) and gonorrhea (*Neisseria gonorrhoeae*) incidence in the USA and explore potential intervention strategies against syphilis. We find that cycles in syphilis incidence can be driven solely by changing sexual behaviour in structured populations. Our model also explains the lack of similar cycles in gonorrhea incidence even if the two infections share the same propagation pathways. Our model similarly illustrates how sudden epidemic outbreaks can occur on time scales smaller than the characteristic demographic time scale of the population and that weaker infections can lead to more violent outbreaks. Behaviour also appears to be critical for control strategies as we found a bigger sensitivity to behavioural interventions than antibiotic treatment. Thus, behavioural interventions may play a larger role than previously thought, especially in the face of antibiotic resistance and low intervention efficacies.

## 1. Introduction

**Figure 1.**
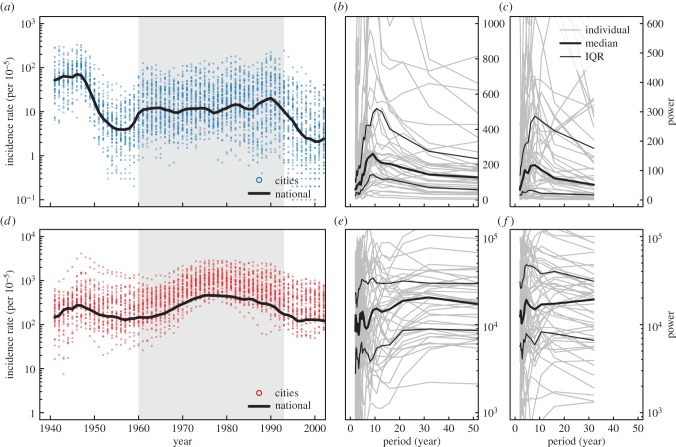
Syphilis and gonorrhea dynamics. (*a*) Syphilis and (*d*) gonorrhea incidence, respectively (per 100 000) as reported to the US Centers for Disease Control (CDC) for the entire USA (black line) and for the largest 62 US cities (points). (*b*,*e*) Are the Fourier power spectra of the cities with complete reports from 1941 to 2002 for syphilis and gonorrhea, respectively; (*c*,*f*) are the Fourier spectra in cases from cities from 1960 to 1993 for syphilis and gonorrhea, respectively. Thick lines report the median power, whereas thin the inter-quartile range (IQR). (Online version in colour.)

It has been hypothesized that syphilis incidence cycles due to host immunity [1]. Recent important work by Grassly *et al*. [1] identified 8–11 year cycles in syphilis incidence (figure 1) and proposed deterministic and stochastic susceptible–infected–recovered (SIR) models to describe dynamics [2]. While the stochastic SIR model exhibited cycles in incidence, evidence for sterilizing immunity after syphilis infection is weak [3–5], and purposefully ignores human sexual behaviour, vast heterogeneities in which have been demonstrated [6]. Additionally, this model does not explicitly consider control of syphilis, both through the use of antibiotics and changes in behaviour [7].

The observed syphilis incidence data (figure 1) do show some evidence of cycling in incidence both from 1941 onward and when restricted to a single generation (1960–1993, as demonstrated by Grassly *et al*.), though the cycles are more striking when observing the period 1960–1993. Thus, if cycles do exist, they may be more likely driven by a change in behaviour beginning in the early 1960s and lasting through the early 1990s [3], than changes in the immunological status of individuals. In this paper, we explore the effects of changing behaviour on sexually transmitted infection (STI) transmission dynamics. Specifically, we model the transmission dynamics of syphilis and gonorrhea (*Neisseria gonorrhoeae*) using a novel, network-based susceptible–infected–susceptible (SIS) model which incorporates adaptive disconnection/reconnection depending on the infectious state of an individual's neighbours [8]. The model illustrates how behaviour can drive cycles in syphilis transmission and not in gonorrhea transmission dynamics, while also describing the evolution of the underlying contact network for transmission. Other effects of adaptive behaviours are discussed, namely self-organized network topology and the relevance of behaviour to epidemic re-emergence. Finally, the model is used to compare the effects of chemotherapy versus behavioural change for syphilis infection control.

## 2. Results

### 2.1. Network model with adaptive behaviour

To examine the impact of behaviour on transmission dynamics, we adapt and expand a previously presented SIS model on adaptive networks [9–11]. Our model examines the effects of long-term behaviour change on epidemic dynamics [12,13]. Briefly, at every time step, every infectious individual transmits the disease to their susceptible neighbours at rate *β*, and recovers to become susceptible again at rate *r*. Meanwhile, every susceptible individual can disconnect from an infectious neighbour at rate *γ*. The network also undergoes its natural growth, independently of the disease, such that individuals can create links at rate *ρ*. These links are created with an individual regardless of its state, but according to a preferential attachment (PA) process where individuals differ in their probability of being chosen. Heterogeneous levels of individual activity can thus be included in the model. Demographics were also considered, through a homogeneous death rate and birth of zero-degree individuals, but its effect on the dynamics appeared minimal. Similarly, different connection schemes were considered—e.g. serosorting, where only susceptibles create links and/or only susceptibles are chosen for links—did not qualitatively change the dynamics. See the Material and methods section for these variations.

**Figure 2.**
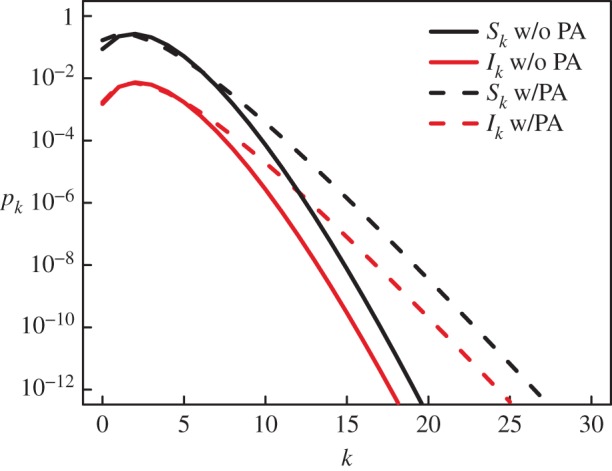
Self-organization of network from adaptative behaviour. Asymptotic degree distribution obtained through the heterogeneous mean-field for both our model and one without PA on networks adapting to a disease with parameters: *r* = 1, *β* = 2/3, *ρ* = 1/50 and *γ* = 1/2. The total degree distribution (*p_k_* = *S_k_* + *I_k_*) has a longer tail than a normal distribution (but is not statistically different (KS Test, *D* = 0.196, *p* = 0.615)) for the case without PA (〈*k*〉 = 2.32) and features an exponential tail (*p_k_* ∝ exp (*−*λ*k*) with *λ* ≈ 1.2, 〈*k*〉 = 2.00) for the case with PA.

We follow the system through a heterogeneous mean-field approximation, where individuals are distinguished by their states (S or I) and by their number of connections (degree *k*). This formalism allows us to describe the evolution of both the epidemics and the underlying network even in the presence of significant heterogeneity [14–18]. The mean-field approximation is given by2.1
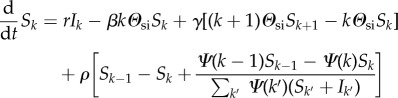
and2.2
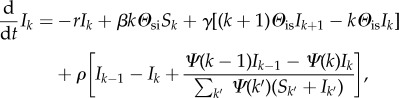
where *Θ*_si_ and *Θ*_is_ are mean-field quantities giving the average probability that a link stemming from a susceptible (infectious) node leads to an infectious (susceptible) node, respectively. They are2.3
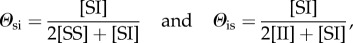
where [*XY*] is the relative density of links joining a node in state *X* with a node in state *Y*. The dynamics governing contacts are given by2.4
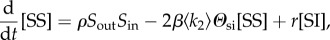
2.5
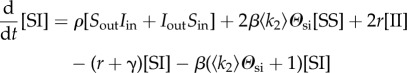
2.6
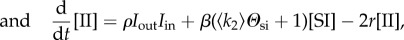
where 〈*k*_2_〉 = ∑_*k*_*k*(*k*−1)*S*_*k*_/∑_*k*_*kS*_*k*_ is the average excess degree of susceptible individuals and *S*_out/in_ (*I*_out/in_) are the probabilities that a new link stems *out* from a susceptible or an infectious individual or reaches *in* a susceptible (infectious) individual; e.g. written *S*_out_ = ∑*_k_S_k_* and *S*_in_ = ∑*_k_Ψ*(*k*)*S_k_*/∑*_k_Ψ* (*k*)(*S_k_* + *I_k_*). In all cases, *Ψ*(*k*) corresponds to the preferential factor given to an individual of degree *k* when choosing a neighbour. When PA is considered, we use *Ψ*(*k*) = *k* + 1; such that a node of degree *k* is chosen with probability proportional to *k* + 1 to allow for nodes of degree zero to also receive new connections. Otherwise, we use *Ψ*(*k*) = 1 for all *k* for a uniform attachment. The resulting network topology is shown in figure 2. With this process, our model can self-organize to reproduce the exponential degree distributions observed in many sexual networks [19]. The goal of this model is to qualitatively reproduce the observed syphilis dynamics and not estimate transmission-relevant parameters, though they are based on the natural history of syphilis and gonorrhea [20,21]. Thus, when comparing to historical data, PA is considered and parameters are chosen to reflect those in the literature rather than through statistical model-fitting. Monte Carlo simulations are conducted on networks of size 20 000 to represent the sexually active population of a population of size 200 000. When only theoretical results are desired, we use uniform attachment and *r* = 1 for simplicity and faster model integration.

### 2.2. Cycles from host behaviour

**Figure 3.**
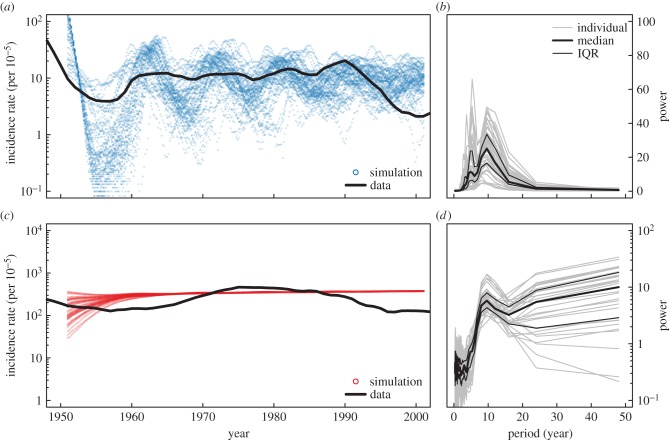
(*a*) Simulated syphilis and (*b*) gonorrhea dynamics. (*a*,*c*) Show 50 simulated syphilis and gonorrhea incidence (per 100 000 points) and observed national data (lines). (*b*,*d*) Show the Fourier power spectra of the simulated time series. Following Grassly *et al*. [1], we take the first difference of our time series before calculating the spectral density with Daniell smoothers of 3 years. For simulated data, we remove the first 5 years of the time series to allow for stochastic burn-in and let the contact network stabilize. For the mean-field approximations, Fourier transforms were taken on the undifferenced time series as the ODEs are smooth and oscillate around a stable mean. Thick lines report the median power, and thin the IQR. Dynamics are similar to observed incidence in US cities. Note, for gonorrhea, the dominant contributions to the power spectra are longer than the observed data window (50 years). The model uses PA and parameters were chosen to reproduce the prevalence and periodicity observed in the data assuming a 79 days average recovery period for syphilis. Full parameters: link creation rate (*ρ*) = 1/10 yr^−1^, disconnection rate (*γ*) = 1/158 d^−1^, transmissibility (*β*) = 1/53 d^−1^, recovery rate (*r*) = 1/79 d^−1^ (syphilis), 1/6.5 yr^−1^ (gonorrhea). Thus, the behavioural parameters reproducing the periodicity were found to be an average disconnection period of the order of five months and an average delay for new connections of the order of 5 years (with wide variations). (Online version in colour.)

**Figure 4.**
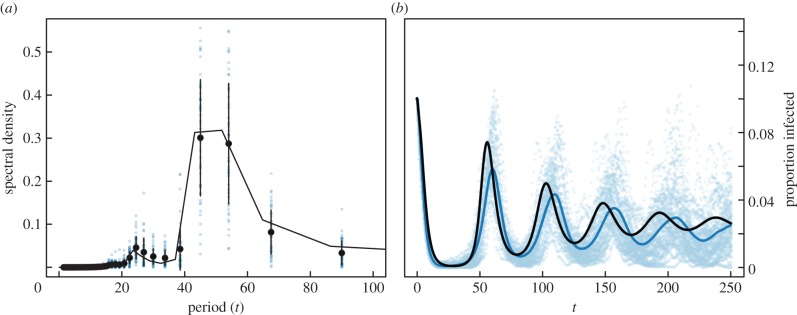
Periodicity in the simulated model and in the heterogeneous mean-field approximations (*a*) Fourier transform of the simulated and integrated time series. The big markers represent the average spectral density for the 50 time series shown on the left side, with error bars consisting of 1 s.d. and outliers shown with smaller markers. The solid line is the spectral density of the Fourier transform of the integrated analytical model. (*b*) Time evolution of the model without PA and with *r* = 1, *β* = 2/3, *ρ* = 1/50 and *γ* = 1/2. The dynamics start with an initially exponential degree distribution {*pk*} (mean degree 〈*k*〉 = 1.8087) and an initial infection *I_k_*(0) = *p_k_*/10 for *I*(0) = ∑*_k_I_k_*(0) = 1/10. The points represent the 50 first time series obtained by Monte Carlo simulation on networks of size 20 000, while the average of 500 such time series is represented by the shaded curve. The black curve is the integration of our model. (Online version in colour.)

Though it has been suggested that cycles in syphilis incidence are due to cycling in immune status of the population [1], evidence for long-lasting acquired immunity to repeat infection remains limited [3–5]. In simulations of our model, we find roughly 10 year cycles in syphilis incidence driven by behaviour (figure 3). As the prevalence of infection rises, individuals are more likely to be connected to an infected individual and thus causing more disconnections globally. As the prevalence decreases there are less infectious individuals and thus new links are more likely to remain. Importantly, these disconnection/reconnection decisions are made locally, and do not depend on the state of the entire system, nor directly on prevalence. Our mean-field formalism is shown to roughly follow the average time evolution of the model and to accurately predict its periodicity (figure 4). Note that although the peak height predicted by the ODEs is decreasing with time (damped oscillator), this is mostly due to the spread between individual realizations as they go out of phase. It is not surprising that the mean-field formalism only approximates the time evolution of the actual model as it has been previously shown that similar dynamics are highly dependent on knowing the state of one's neighbours [11]. Consequently, a more compartmentalized model would be more accurate; such as the one first published by Marceau and colleagues [11,18,22] where nodes are distinguished by their state, degree and number of infectious neighbours (i.e. *S_k_*_,*i*_ and *I_k_*_,*i*_). However, the current model is much simpler and accurate enough for the purpose of this work. We thus hereafter concentrate on analytical results rather than Monte Carlo simulations.

**Figure 5.**
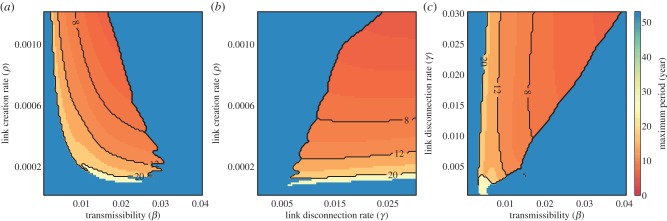
Simulated dynamics hold over a broad range of realistic parameters. (*a*–*c*) Heatmaps of the maximum value of the Fourier spectrum for a given set of parameter values. Fourier spectrum taken on the undifferenced time series (see Material and methods). (*a*) Variation in link creation rate (*ρ*) and transmissibility (*β*), (*b*) shows link creation (*ρ*) and disconnection rate (*γ*) and (*c*) shows link disconnection rate (*γ*) and transmissibility (*β*). Contours indicate periodicities of 8, 12 and 20 years. Figure shows dynamics are more influenced by behaviour than biology. Baseline parameters identical to figure 3.

We find long-period cycles over broad ranges of parameters, and a strong influence of link creation rate on the observed dynamics (figure 5). Figure 5 also shows the relative contribution of behavioural change (link creation/disconnection rates) to biological change (transmissibility). We find changes in link creation rate to be at least as effective in changing the cycle length as changes in transmissibility (figure 5*a*). Halving transmission from 0.02 to 0.01 (with constant link creation) increases the periodicity from 7.4 to 10.4 years, which is the same effect as halving the link creation rate from 0.0006 to 0.0003 (with constant transmission). Halving link creation from 0.00024 to 0.00012 (with transmission = 0.02) increases the periodicity from 13 to 26 years, whereas halving transmission from 0.008 to 0.004 (with link creation = 0.0006) increases the periodicity from 10.4 to 17.4 years.

Observed transmission dynamics differ markedly between syphilis and gonorrhea. Gonorrhea incidence shows no dominant peaks in the Fourier spectra for either 1941–2002, or the generation 1960–1993 (figure 1). This is either evidence for no cycling or a cycle whose period is greater than the observed window of data [23]. Our model captures this lack of periodicity for gonorrhea. Syphilis induces greater sequelae than gonorrhea [3,24], and thus there are less asymptomatic infections. This suggests two assumptions: we assume gonorrhea has a longer infectious period and does not influence changes in network topology. That is, an individual's probability of disconnection is driven by their partner's infection with syphilis, and not gonorrhea. This is reflected in differences in reporting rates between gonorrhea and syphilis [25], particularly exhibiting a lack of saliency in reporting gonorrhea [26]. Thus, this lack of periodicity in gonorrhea is a result of its longer characteristic time scale which offers robustness to short behavioural cycles in the host population. Our results are similar when using an infectious period of gonorrhea three times smaller.

### 2.3. Epidemic re-emergence of weak infections

Interestingly, our model features a universal lack of an epidemic threshold in the limit of infinite population size. This complements earlier work [27]. That is, for any initial condition of the network and any biological parameters of the disease, every transmissible pathogen (non-zero value of the transmissibility parameter) corresponds to a non-zero epidemic steady state. Even if the disease is at first incapable of spreading throughout the population, links will systematically be created until the network topology is dense and heterogeneous enough to initiate spread. This has been observed in several countries where syphilis incidence is low and behavioural change eventually led to severe outbreaks. For instance, in the French Caribbean islands where low incidence in the 1990s led to the cancellation of mandatory notification programmes, while social precariousness led to increase in epidemic potential and re-emergence of syphilis [28,29].

In the case of realistic population sizes, diseases with low transmissibility will experience epidemic re-emergence. Our model explains why diseases may repeatedly survive at very low number of infected individuals before re-emerging and subsequently disappearing again when individuals start disconnecting. This phenomenon may be unsurprising considering our model can be considered as a variation on the work of Zhou *et al.* [9] which also featured epidemic reemergence. However, our model shows how assumptions previously thought necessary for epidemic re-emergence are in fact superfluous: the introduction of new nodes (demographics) and isolation avoidance (enforcing all individuals to maintain at least one contact at all times). Removing the first illustrates how epidemic cycles and re-emergence can occur on time scales much smaller than the characteristic demographic scale [30], and the second is especially relevant in the context of cycles in STIs, where there is no requirement that every member of a population must maintain at least one sexual partner at all times [6].

**Figure 6.**
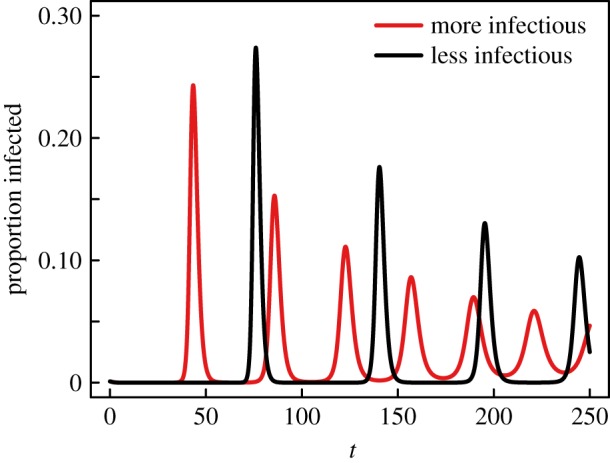
Smaller transmissibility leads to rarer, but larger outbreaks. Epidemic re-emergence obtained through integration of the model for two similar diseases on a network where all nodes are initially of degree 4 and of which a fraction 0.001 is infectious. For the red curve, the dynamics use *r* = 1, *β* = 3/10, *ρ* = 1/10 and *γ* = 17/14. For the black curve, the dynamics are almost identical, but slightly less transmissible: using *β* = 2/10. Both cases consider uniform attachment.

With this phenomenon of epidemic re-emergence, our model also generalizes the notion of building epidemic potential through the recruitment of susceptibles [31]. Our model builds epidemic potential through individual behaviour, potentially explaining epidemic bursts occurring on short time scales. Interestingly, unlike demographics, this build-up of epidemic potential is significantly coupled with the disease dynamics, leading to interesting effects caused by adaptive behaviour. For instance, we can illustrate how, in the context of epidemic bursts, lower transmissibility does not imply smaller outbreaks. Consider two diseases subject to similar dynamics, with one being slightly less transmissible than the other (*β*_2_ < *β*_1_). If the difference is small enough, we can expect the two diseases to have a similar equilibrium around which they will both cycle. Consider now that both diseases are approaching this equilibrium from the higher values of *I*_1_(*t*) and *I*_2_(*t*) (i.e. from ‘above’). The network will be able to push the less virulent disease to a lower fraction of infectious individuals (i.e. *I*_2_(*t*) < *I*_1_(*t*)). In return, this implies that the network will be able to create more links before *I*_2_(*t*) gets to its equilibrium than it can for *I*_1_(*t*), as the second disease has now both lower incidence and is transmitted slower than the first. These additional links can then be translated into more infections during the epidemic cycles. In short, although the disease with a slightly smaller transmission rate will oscillate more slowly than the other, it will do so by featuring more violent epidemic re-emergence (figure 6).

### 2.4. Epidemic control

The CDC has outlined treatment guidelines [32] for syphilis that include parenteral injection with penicillin G or oral macrolides or tetracyclines which increase clearance of the spirochete. They have also launched a programme to eliminate syphilis in the USA [7]. The Syphilis Elimination Effort (SEE) has three major threads: enhancement of public-health services, evidence-based interventions that are culturally appropriate and accountability [33]. A major component of the plan is ‘the development and testing of effective biomedical and behavioural interventions to reduce syphilis transmission…’ [7, Chapter 4, p. 13]. In the face of increasing antibiotic resistance in syphilis [34], it may be worth the added effort of implementing behavioural interventions preferentially.

Here we explore treatment of syphilis with both antibiotics (chemotherapy) and through behavioural interventions. We assume chemotherapy acts through reducing transmissibility and increasing recovery rate and behavioural interventions act through changing the probability of an individual severing or creating contacts. In both cases, we refer to the ‘efficiency’ of the intervention as the percentage change in the focal parameter (transmissibility, recovery rate, disconnection/reconnection probability). Examples of behavioural interventions considered here would include partner notification (disconnection initiated from the uninfected), abstinence and monogamy education [35], all key interventions in the CDC's SEE program [7].

**Figure 7.**
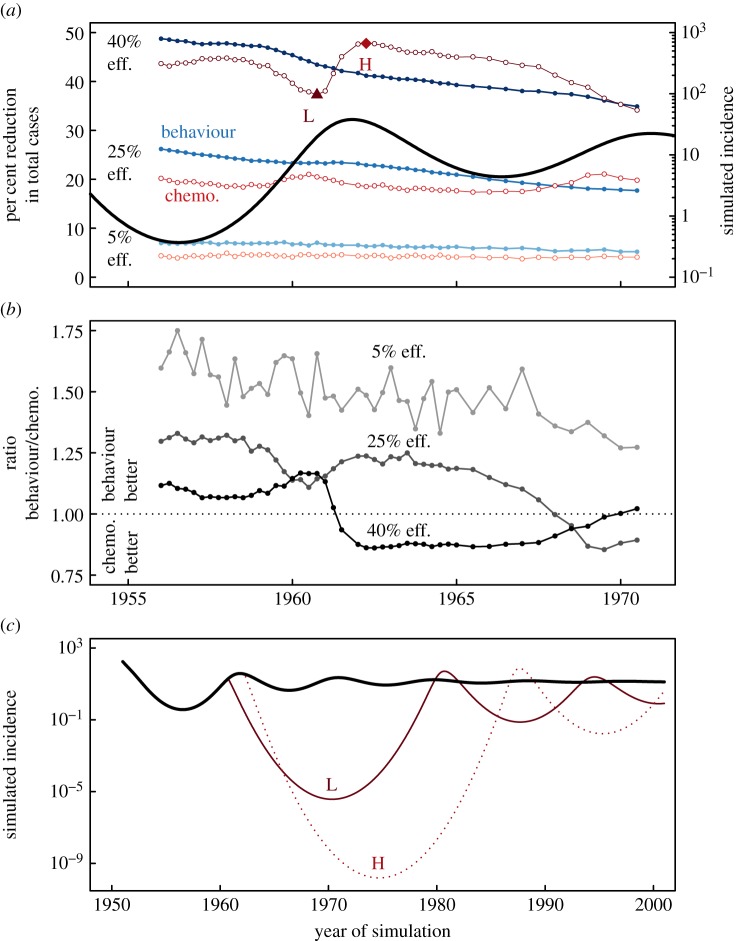
Chemotherapy and behavioural interventions exhibit different efficiencies. (*a*) Shows the per cent reduction in total cases of behavioural (filled markers, in blue) and chemotherapy interventions (open markers, in red) from a no-treatment scenario initiated with various efficiencies (as a per cent reduction in either transmissibility or link creation or severing) at various times in an epidemic. Thick black line is the mean-field ODE results. Simulated incidence is per 100 000. (*b*) Shows the ratio of efficacies of behavioural interventions to chemotherapy interventions (percentages from (*a*)) initiated at different time points in the epidemic. (*c*) Shows example time series of chemotherapy interventions initiated before and after the crest of the epidemics (positions marked as ‘H’ and ‘L’ in (*a*)). We see differences in the effectiveness of interventions depending on the efficiency and timing. Dynamics and parameters identical to figure 3. (Online version in colour.)

For low levels of intervention efficacy (approx. 5%), we find behavioural interventions to be between 27% and 75% more effective than chemotherapy, depending on the time of initiation (figure 7). For higher levels of intervention efficacy, we find behaviour to be more effective if interventions are begun early. However, we find chemotherapy to become more effective as intervention is delayed until it can capture the ‘momentum’ of the epidemic cycles. More precisely, this happens just after the maximum of a given epidemic cycle where chemotherapy reduces the spread of the disease, while the intervention still benefits from the natural adaptive behaviour of the population.

Consequently, we find relatively large differences in the effectiveness of chemotherapy depending on the state of the epidemic (rising incidence or falling incidence) when the intervention is initiated. Figure 7*c* shows three example time series with and without chemotherapy treatment. Treatment initiation before the crest of the epidemic is 23% less effective than treatment after the crest (37% reduction in total cases versus 48% reduction). Additionally, the simulated incidence after treatment is 4.2 × 10^5^ times lower when treating after the crest than treatment before (1.6 × 10^−10^ versus 3.7 × 10^−6^) increasing the odds of eradication. This unrealistically small incidence is due to the continuous nature of the mean-field approximation, but can be directly translated to a stochastic probability of eradicating the pathogen. It is important to note that we find behavioural interventions are less influenced by the timing of initiation, which becomes important under imperfect surveillance.

## 3. Discussion

We have presented an adaptive network model of disease transmission and behaviour to explain the observed cycles in syphilis. We found that dynamic rewiring of an individual's sexual contacts alone can cause epidemics to wax and wane. Our simple model recreates the dynamics of both syphilis and gonorrhea over similar time periods and broad ranges of realistic parameters (including incorporating demography and serosorting behaviour), and recreates observed distributions of sexual partners. Furthermore, our model illustrates how sudden epidemic bursts can occur on short time scales and how weaker infections (smaller transmissibility) can actually lead to larger, but rarer, epidemic peaks. This work further highlights the importance of including human behaviour when examining the transmission dynamics of STIs.

Following the CDC's national SEE, we examined two forms of syphilis control—chemotherapy with antibiotics and behaviour modification—and found differences in effectiveness depending on the timing of treatment initiation. In the light of the global rise in antibiotic resistance of *T. pallidum* to macrolides and tetracyclines [34], and the relative difficulty of successful behavioural interventions [36], the CDC acting through public-health departments must strike a balance between effectiveness of intervention and the risk of potential antibiotic failure in the future. Importantly, behavioural interventions may play a larger role than previously thought, especially when intervention efficacies are low. We have presented the effectiveness of *relative* changes in transmission in response to behaviour or chemotherapeutic interventions, but it should be noted that due to the difficulties in implementation, the costs of say, a 25% reduction in acquisition of new partners may not be equivalent to a 25% increase in treatment. Additionally, behavioural interventions are often difficult to implement in high-risk populations (e.g. intravenous drug-users), but nonetheless should be considered. Detailed cost–benefit analyses should be conducted, and we leave this for future research.

Our model is relatively simple and was chosen to favour parsimony over detail [37]. Many details of syphilis and gonorrhea biology and human behaviours were simplified. Despite this, qualitative predictions are possible. Specifically, we focused solely on primary and secondary syphilis infection, though latent syphilis is not transmissible [32], and tertiary syphilis incidence is very low [3], and we limited behaviour to a simple rewiring probability dependent on the immune status of one's partner. Further work could expand the model to include more detailed behaviour mechanisms (e.g. considering demographic dynamics, disconnection unrelated to epidemiological status or heterogeneity in personal tolerance to a contact's infection status) and a more systematic exploration of treatment strategies should be undertaken.

Despite these limitations, our simple model captures the dynamical consequences of including a natural simplification of human behaviour, namely that individuals disconnect from those who are infected. This is important given that vast changes in human behaviour over the course of the twentieth century have shaped the dynamics of syphilis in the USA. With concentrated effort and a balance of chemotherapy and behaviour change, the twenty-first century may see its eradication.

## 4. Materials and methods

### 4.1. Empirical data

Primary and secondary syphilis and gonorrhea cases and incidence rates (cases per 100 000 population) from 1941 to 2002 were obtained from to the US Centers for Disease Control and Prevention. We consider the data to be reliable, though it is worth noting that gonorrhea infection tends to be non-specific in men and women, and it may be that chlamydia infection has contaminated the gonorrhea case numbers.

### 4.2. Testing different modelling assumptions

The only necessary feature of the model is the possible disconnection of links that connect a susceptible individual to an infectious individual. While other assumptions have been considered, all were removed to maintain the simplest possible model. Our aim was to gain insights through the qualitative reproduction of the observed data.

As we focused over a long-time horizon, we considered population demographics through a homogeneous death rate for all individuals regardless of their state, and with birth of degree-zero individuals at a rate fixed to maintain a stable population size. We also considered different schemes of creating new links: only susceptible individuals are allowed to create and/or receive new links (serosorting behaviour). As shown in the electronic supplementary material, none of these possible variations yield significant variations in the predicted dynamics for syphilis and are consequently ignored in the main text.

### 4.3. Spectral analysis

Following Grassly *et al*. [1], we take the first difference of our time series before calculating the spectral density with Daniell smoothers of 3 years. For simulated data, we remove the first 5 years of the time series to allow for stochastic burn-in and let the contact network stabilize. For the mean-field approximations, Fourier transforms were taken on the undifferenced time series as the ODEs are smooth and oscillate around a stable mean.

## Supplementary Material

Variations on the model
