# Supplementary figures and images for: Epidemic cycles driven by host behaviour
Source: J R Soc Interface. 2014 Oct 6;11(99):20140575. doi: 10.1098/rsif.2014.0575 (PMC4235258; doi:10.1098/rsif.2014.0575)

Proportion Infected

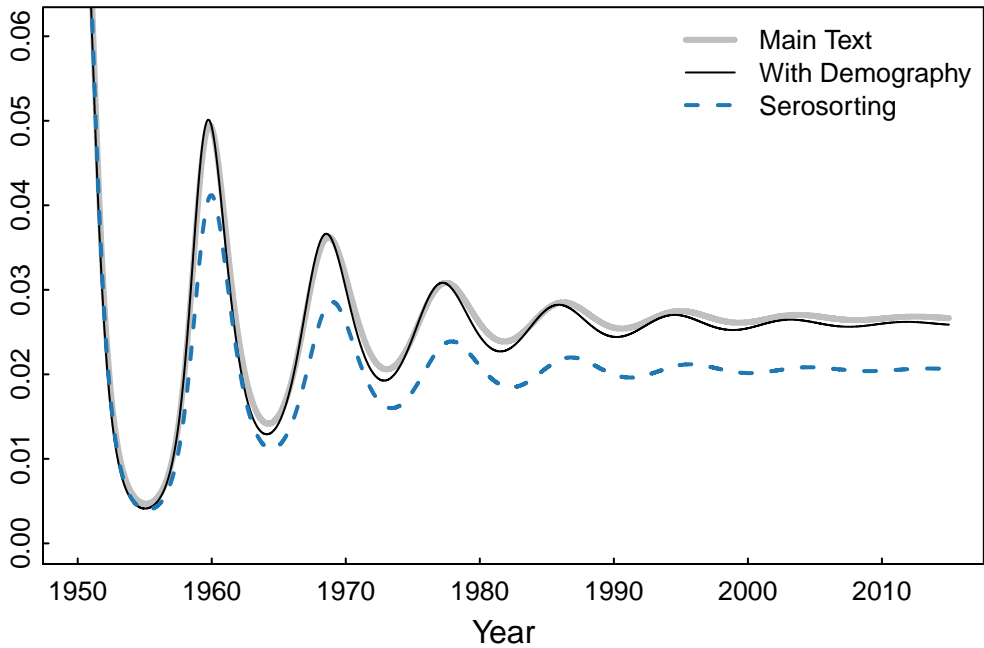

Supplement: Variations on the model [file rsif20140575supp1.pdf]
